# Supplementary material for: Epigenetic modulation of immune synaptic-cytoskeletal networks potentiates γδ T cell-mediated cytotoxicity in lung cancer
Source: Nat Commun. 2021 Apr 12;12:2163. doi: 10.1038/s41467-021-22433-4 (PMC8042060; doi:10.1038/s41467-021-22433-4)
Supplement: Supplementary file 1 — Supplementary Information [file 41467_2021_22433_MOESM1_ESM.pdf]

Supplementary Figures

a

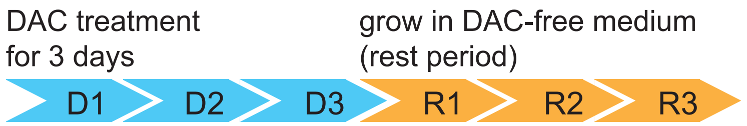

		A549	
		D3	D3R3
Total	Proteins*	1771	2389
	Peptides	17166	26735
Surface**	Proteins*	666	831
	Peptides	8791	11898

* with Q-value < 0.01 and unique peptides ≥ 2
** Uniprot annotation as plasma membrane proteins

b

		H1299		CL1-0	
		D3	D3R3	D3	D3R3
Total	Proteins*	1943	1770	1742	1885
	Peptides	18981	19010	16249	18114
Surface**	Proteins*	661	632	584	622
	Peptides	8562	8741	6902	7590

* with Q-value < 0.01 and unique peptides ≥ 2
** Uniprot annotation as plasma membrane proteins

Supplementary Fig. 1 Summary of the identified plasma membrane proteomes. a Numbers of identified total and surface proteins/peptides in A549 human lung cancer cells subject to 100 nM decitabine (DAC) daily treatment for 72 hours (D3), followed by growing in drug-free medium for three days (D3R3). **b** Numbers of identified total and surface proteins/peptides in

H1299 and CL1-0 human lung cancer cells subject to 100 nM decitabine daily treatment for 72 hours (D3), followed by growing in drug-free medium for three days (D3R3).

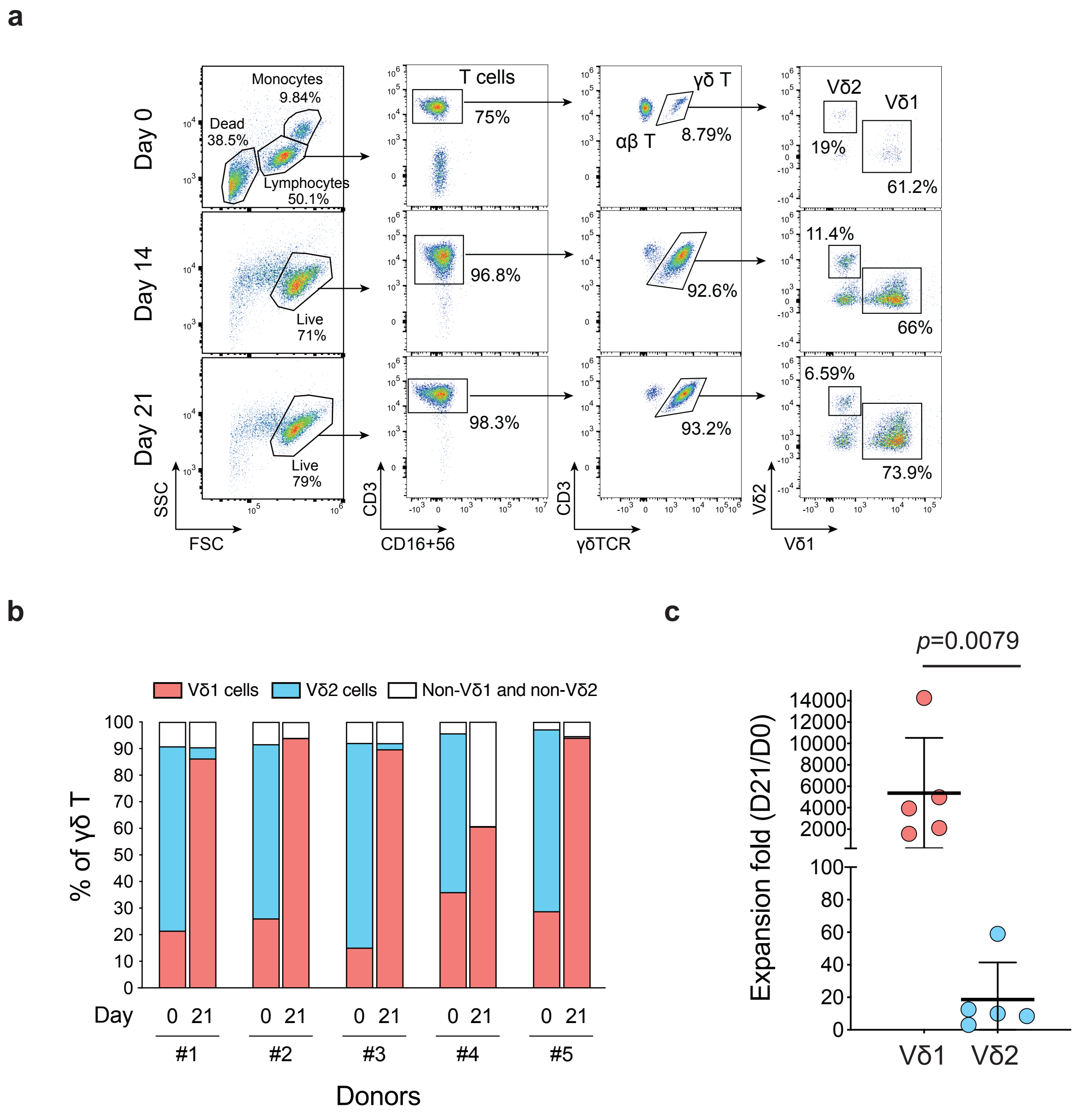

Supplementary Fig. 2 ***Ex vivo*** **expansion of human Vδ1-enriched γδ T cells. a** A representative plot of flow cytometric analysis showing *ex vivo* expansion of γδ T cells from peripheral blood mononuclear cells of a healthy donor on day 0, 14, and 21. FSC: forward

scatter. SSC: side scatter. **b** Bar graphs showing percentages of γδ T cell subsets (i.e., Vδ1, Vδ2, non-Vδ1 non-Vδ2 cells) in the total γδ T populations from the peripheral blood of five healthy donors following *ex vivo* expansion on day 0 and 21. **c** Comparison of expansion folds between Vδ1 and Vδ2 cells from the five donors using our clinical-grade expansion protocol. The *p* value was calculated by the two-sided Mann-Whitney test (n=5, mean ± SD).

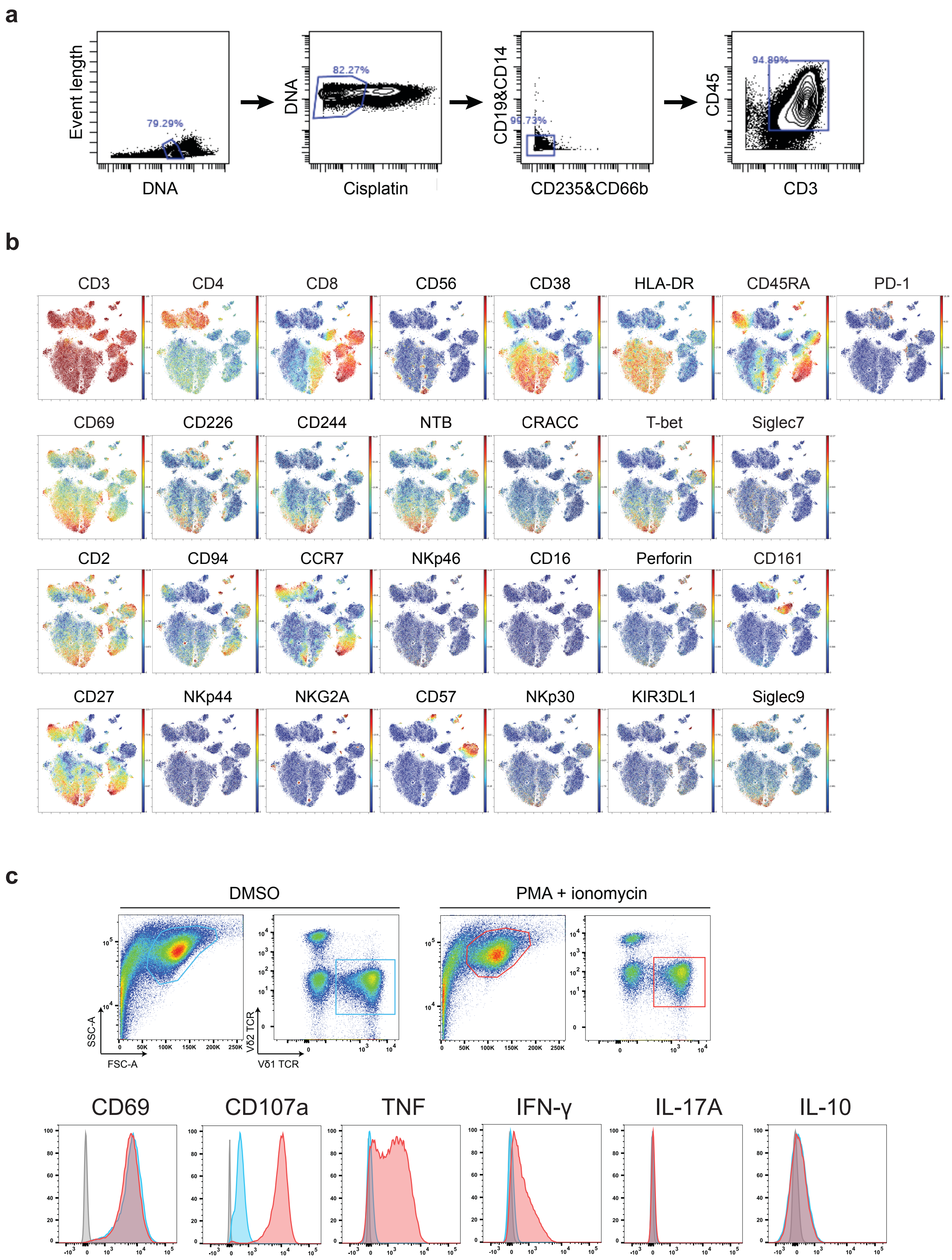
a
Event length
DNA
79.29%
DNA
Cisplatin
82.27%
CD19&CD14
CD235&CD66b
CD45
CD3
94.89%
b
CD3
CD4
CD8
CD56
CD38
HLA-DR
CD45RA
PD-1
CD69
CD226
CD244
NTB
CRACC
T-bet
Siglec7
CD2
CD94
CCR7
NKp46
CD16
Perforin
CD161
CD27
NKp44
NKG2A
CD57
NKp30
KIR3DL1
Siglec9
c
DMSO
PMA + ionomycin
SSC-A
FSC-A
Vδ2 TCR
Vδ1 TCR
CD69
CD107a
TNF
IFN-γ
IL-17A
IL-10

Supplementary Fig. 3 Single-cell mass cytometry analysis of CD3+ T cells before and after the expansion of γδ T cells. a Representative manual gating strategy to identify CD3+ T cells. Doublet and cell debris were gated out by event length and DNA content. Dead cells were then gated out based on the intensity of cisplatin staining. B cells, monocytes, granulocytes and red blood cells were excluded using CD19 and CD14, CD235 and CD66b. Finally, CD3+ T cells were defined by the level of CD3 expression. **b** An extended panel for immunophenotyping PBMCs at baseline and after *ex vivo* expansion. Each dot represents a single cell, and the color of each dot represents the expression level of the indicated markers for each tSNE plot. Red is high, and blue is low. **c** Flow cytometric analysis of T cell activation markers (CD69, CD107a), antitumor effector cytokines (TNF, IFN-γ) and protumor effector cytokines (IL-17A, IL-10) produced by Vδ1 cells following phorbol 12-myristate 13-acetate (PMA, 30 ng/ml) and ionomycin (1 μg/ml) stimulation for 4 hours. DMSO: Dimethyl sulfoxide. FSC: forward scatter. SSC: side scatter.

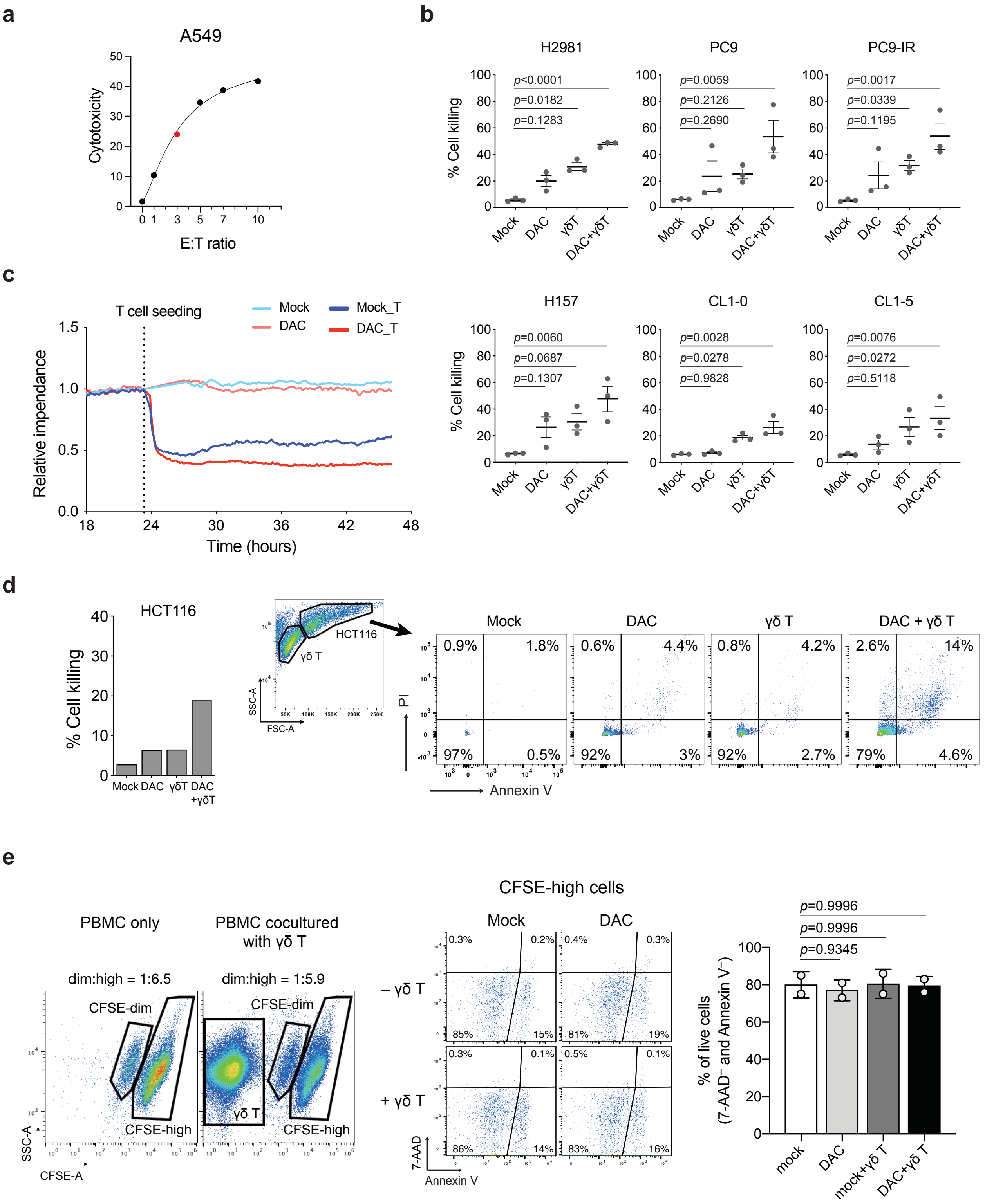

Supplementary Fig. 4 Decitabine-synergized cytotoxicity of cancer cells by γδ T. a Cytolysis of A549 lung cancer cells by γδ T cells at an effector to target (E:T) ratio of 1, 3, 5, 7, and 10 is

determined by calcein-release assay. **b** Annexin V and propidium iodide apoptosis assays of human lung cancer cell lines — H2981, PC9, PC9-IR, H157, CL1-0, and CL1-5 — upon treatments with 100 nM decitabine (DAC) alone, γδ T cells alone or DAC/γδ T cells combination. E: T ratio is 3:1. Data from three biological replicates are presented as mean ± SEM. The *p* value is calculated by one-way ANOVA with Tukey's multiple comparison test. **c** Real-time impedance-based cell viability measurement of A549 lung cancer cells subject to mock, DAC alone, γδ T alone or combination of DAC and γδ T treatment using the electric cell-substrate impedance sensing (ECIS) system. Acquired raw data are normalized by the impedance at the latest time prior to adding γδ T cells of each condition. DAC treatment alone has minimal effects on cell viability but may potentiate γδ T-mediated cytolysis when combined with γδ T. **d** Bar graphs showing annexin V and propidium iodide (PI) apoptosis assays of HCT116 colorectal cancer cells upon treatments with 100 nM DAC alone, γδ T cells alone or DAC/γδ T cells combination. Representative flow cytometric analysis is shown on the right. **e** Annexin V and 7-AAD apoptosis assays in the peripheral blood mononuclear cells (PBMCs) from a healthy donor upon treatments with 100 nM DAC alone, γδ T cells alone or DAC/γδ T cell combination. The effector to target (E:T) ratio is 3:1. Bar graphs showing annexin V and 7-AAD apoptosis assays in PBMCs from two healthy donors. Data are presented as mean ± SD. The *p* value is calculated by nonparametric one-way ANOVA test. CFSE: Carboxyfluorescein succinimidyl ester, used as a live cell dye for PBMCs.

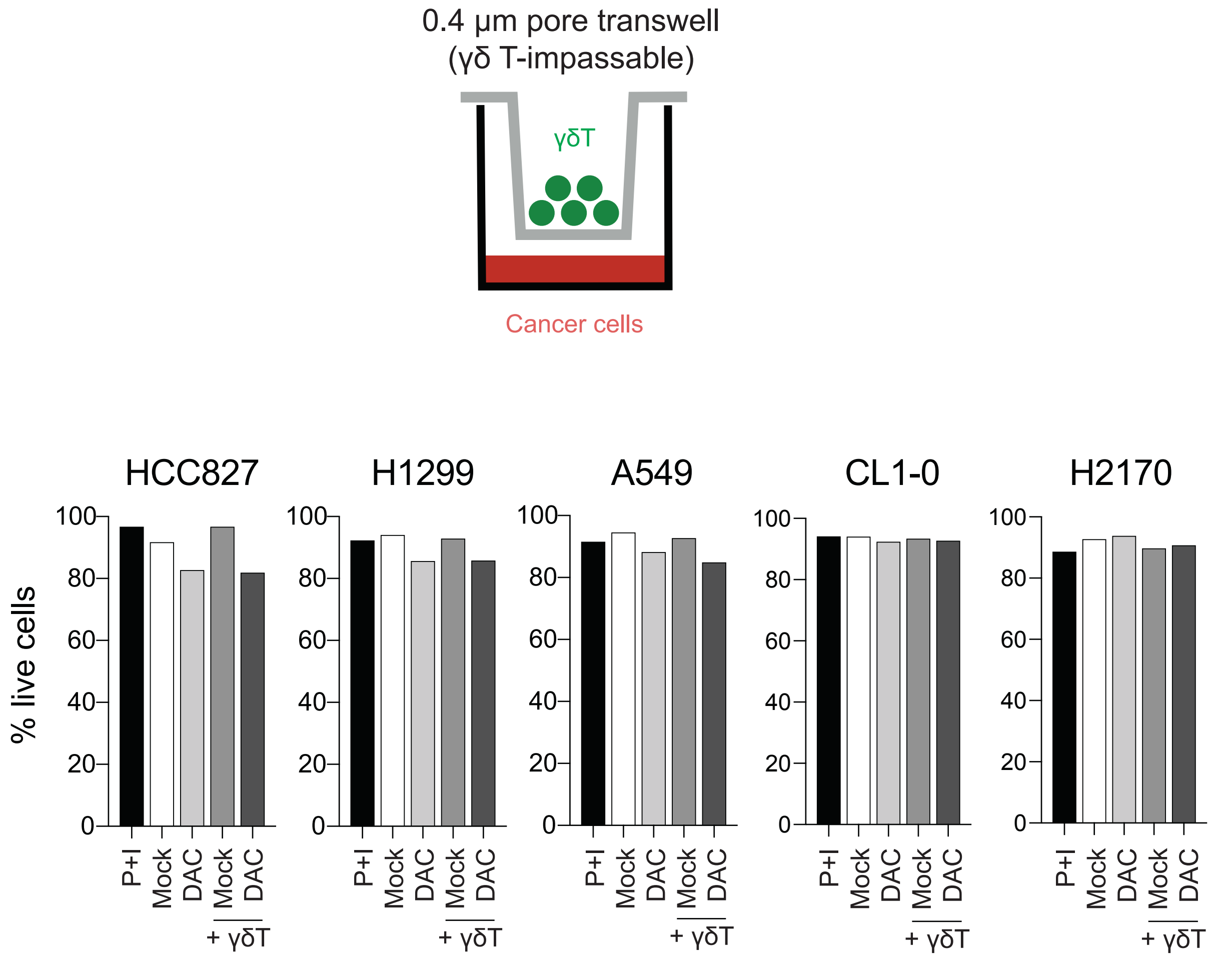

Supplementary Fig. 5 Non-contact killing assays of five lung cancer cells by γδ T cells in a Transwell coculture system permeable to cytokines and cytotoxic mediators. Lung cancer cell lines are pretreated with DAC for 72 hours and rest for 3 days before coculture with γδ T cells. Cell death is analyzed by annexin V apoptosis assays after coculture for 24 hours. The experiment for each cell line was performed once and independently. P+I: PMA (phorbol 12-myristate 13-acetate) and ionomycin.

a

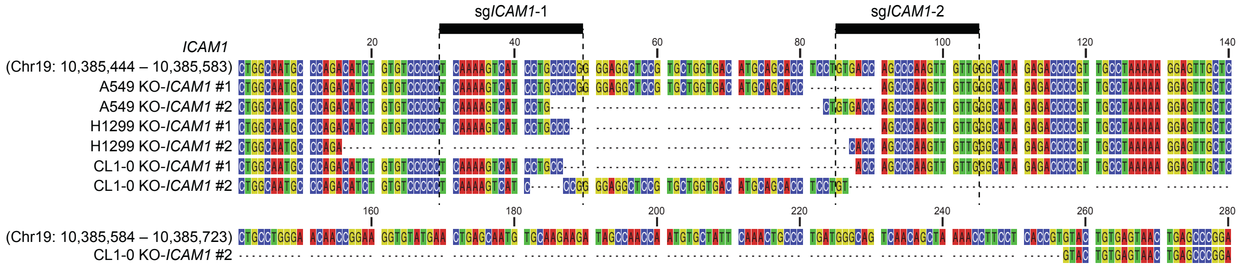

b

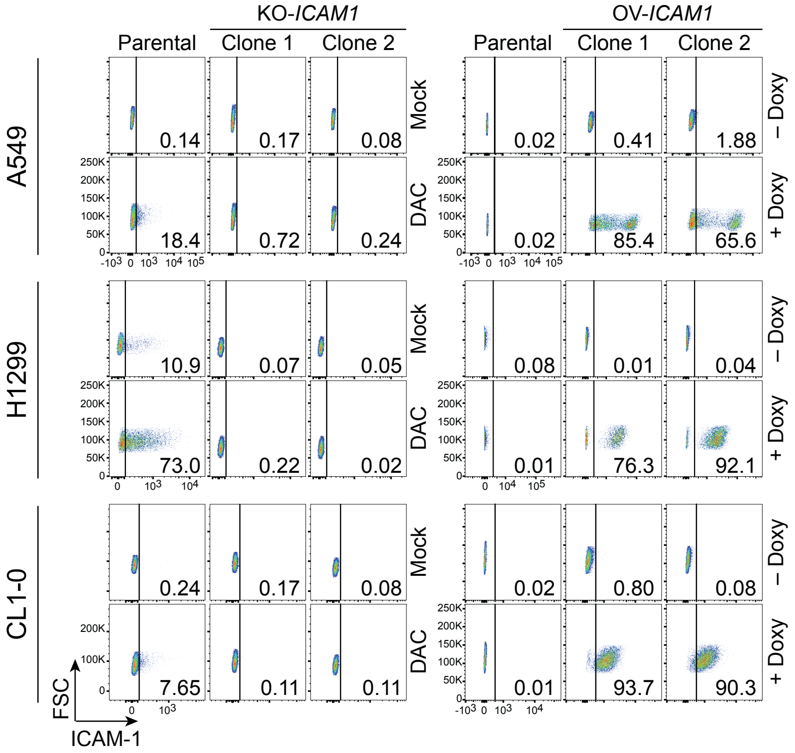

Supplementary Fig. 6 Validations of *ICAM1* genomic sequence and surface ICAM-1 expression of lung cancer cells with *ICAM1*-depletion or -overexpression. a Sanger sequencing validation of the CRISPR/Cas9-edited *ICAM1* genome locus of lung cancer cells. Multiple sequence alignments of the KO-*ICAM1* cells against the reference *ICAM1* genome locus. Alignment gaps are indicated with a hyphen (-) to represent the lost (knockout) regions of the edited *ICAM1* genome locus. **b** Flow cytometric analysis of lung cancer cells subject to

CRIPSR knockout of *ICAM1* (KO-*ICAM1*, left panels) or overexpression of *ICAM1* using a doxycycline (Doxy)-inducible Tet-on system (OV-*ICAM1*, right panels). Two independent clones of each genetic manipulation for A549, H1299, and CL1-0 cell lines are shown. x-axis: signal intensities of ICAM-1. y-axis: forward scatter (FSC). Gating strategies are shown in Supplementary Fig. 16.

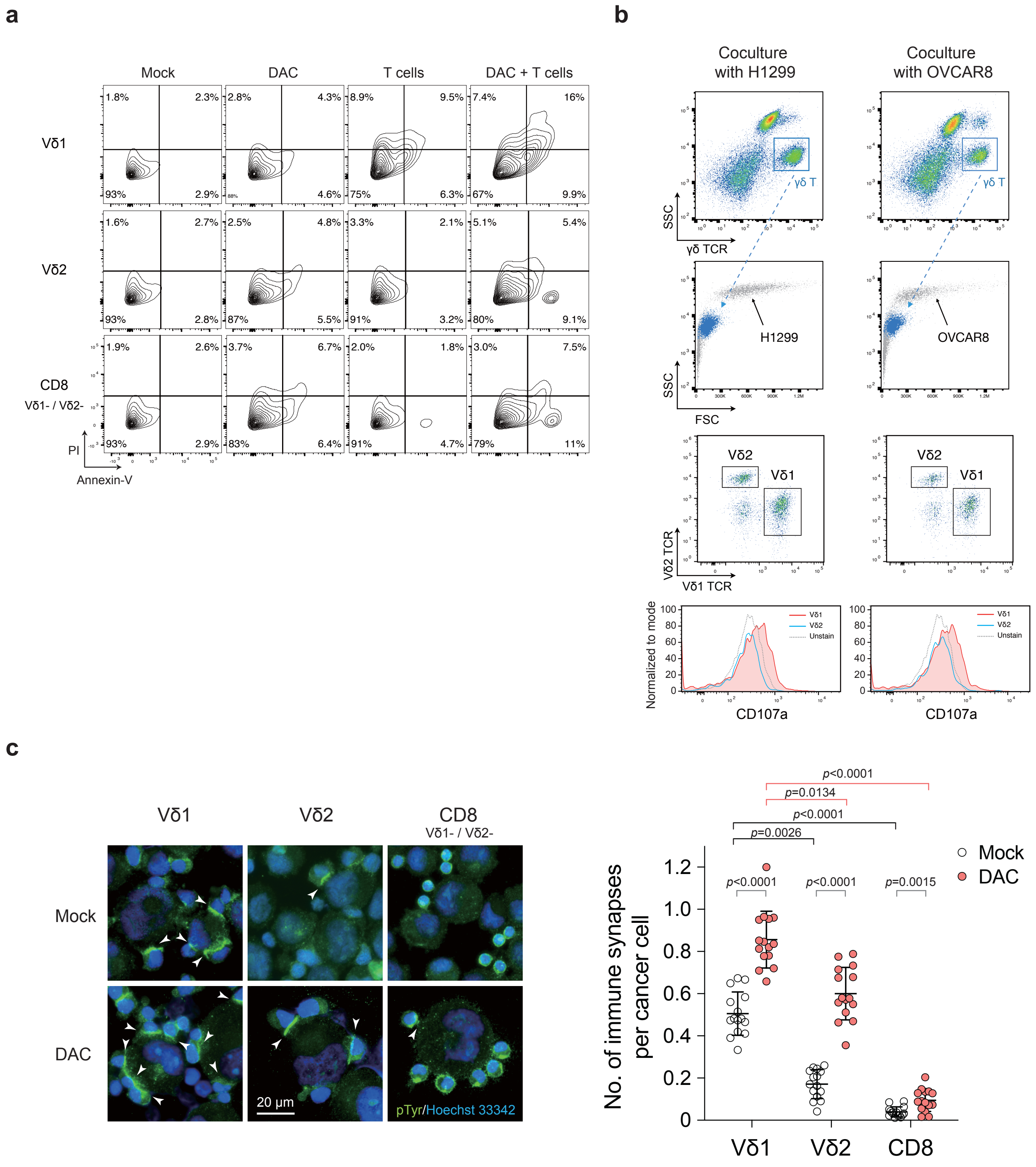

Supplementary Fig. 7 Cell-mediated cytotoxicity, CD107a expression, and immune synapse formation of sorted Vδ1, Vδ2, or CD8 T cells in coculture with human cancer cells. a Flow cytometric analysis of Annexin V and propidium iodide (PI) apoptosis assays in H1299 human lung cancer cells upon treatments with phosphate-buffered saline (Mock), 100 nM decitabine

(DAC) alone, T cells (i.e., Vδ1, Vδ2 or CD8) alone or DAC/T cell combination. Gating strategies are shown in panel b. The effector to target (E: T) ratio is 3:1. The data demonstrate that Vδ1 cells possess relatively higher cytolytic capacity compared with Vδ2 or CD8+ T cells. **b** *Upper,* Representative manual gating strategy to identify Vδ1 and Vδ2 cell populations. FSC: forward scatter. SSC: side scatter. *Lower*, Flow cytometric analysis of CD107a expression on Vδ1 and Vδ2 cells after coculture with H1299 human lung cancer (*left*) or OVCAR-8 human ovarian carcinoma cells (*right*) for 5 hours at an E: T ratio = 1:1. The data show that Vδ1 cells expressed higher levels of CD107a after coculture with lung cancer cells. **c** *Left,* Representative immunofluorescence images of immune synapses (pTyr staining) between T cells (i.e., Vδ1, Vδ2 or CD8) and H1299 cells pretreated with PBS (Mock) and DAC. Arrows denote immune synapses between T and H1299 cells. Scale bar: 20 µm. *Right,* Dot plots showing numbers of immune synapses per cancer cell on fifteen randomly taken high power fields for H1299 cells pretreated with PBS (Mock) or DAC and cocultured with Vδ1, Vδ2, and CD8+ T cells (mean ± SD). The *p* value is calculated by Welch's t test (two-sided, Mock vs. DAC) or Kruskal-Wallis test with Dunn's multiple comparisons test (Vδ1 vs. Vδ2 vs. CD8)

a

b

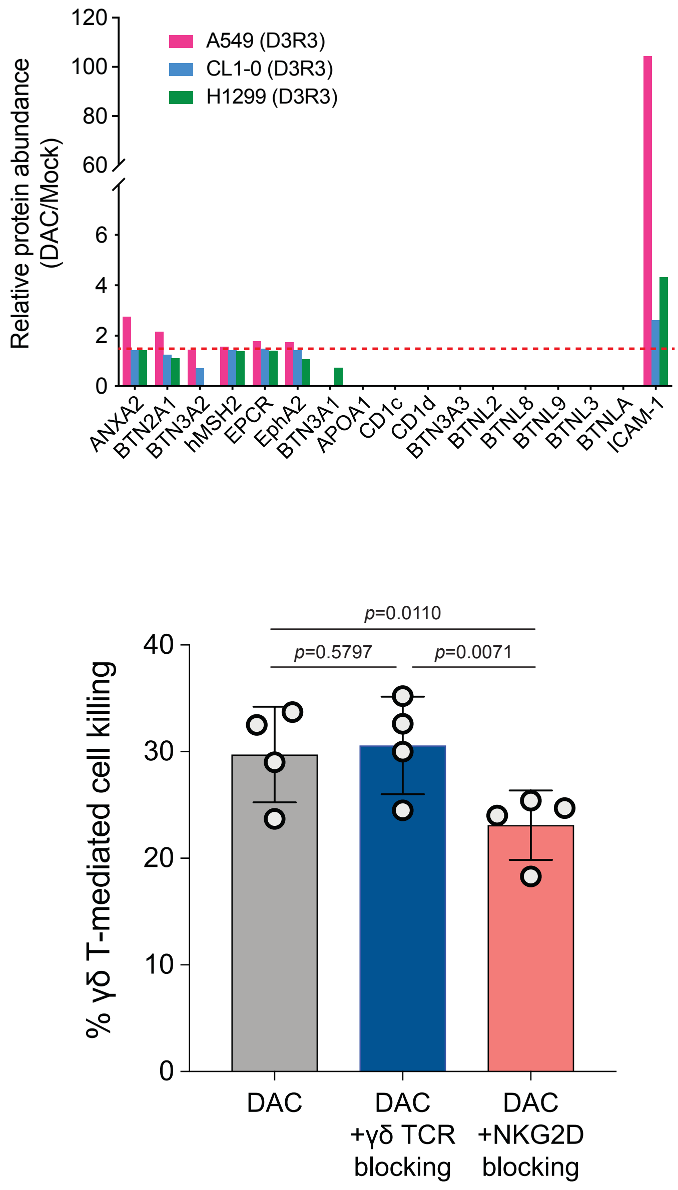

Supplementary Fig. 8 Blocking of NKG2D attenuates γδ T cell-mediated cytolysis of lung cancer cells following decitabine treatment. a Bar graphs showing relative protein abundance of putative ligands for γδ TCR in surface proteomes of A549, H1299 and CL1-0 cells following decitabine (DAC) treatment at D3R3 as compare with mock-treated cells. **b** Bar graphs showing γδ T-mediated cytotoxicity of human H1299 lung cancer cells measured by annexin V and

propidium iodide apoptosis assays after blocking of γδ TCR or NKG2D. Data are summarized from three independent experiments and presented as mean ± SD. The *p* value was calculated by one-way ANOVA test.

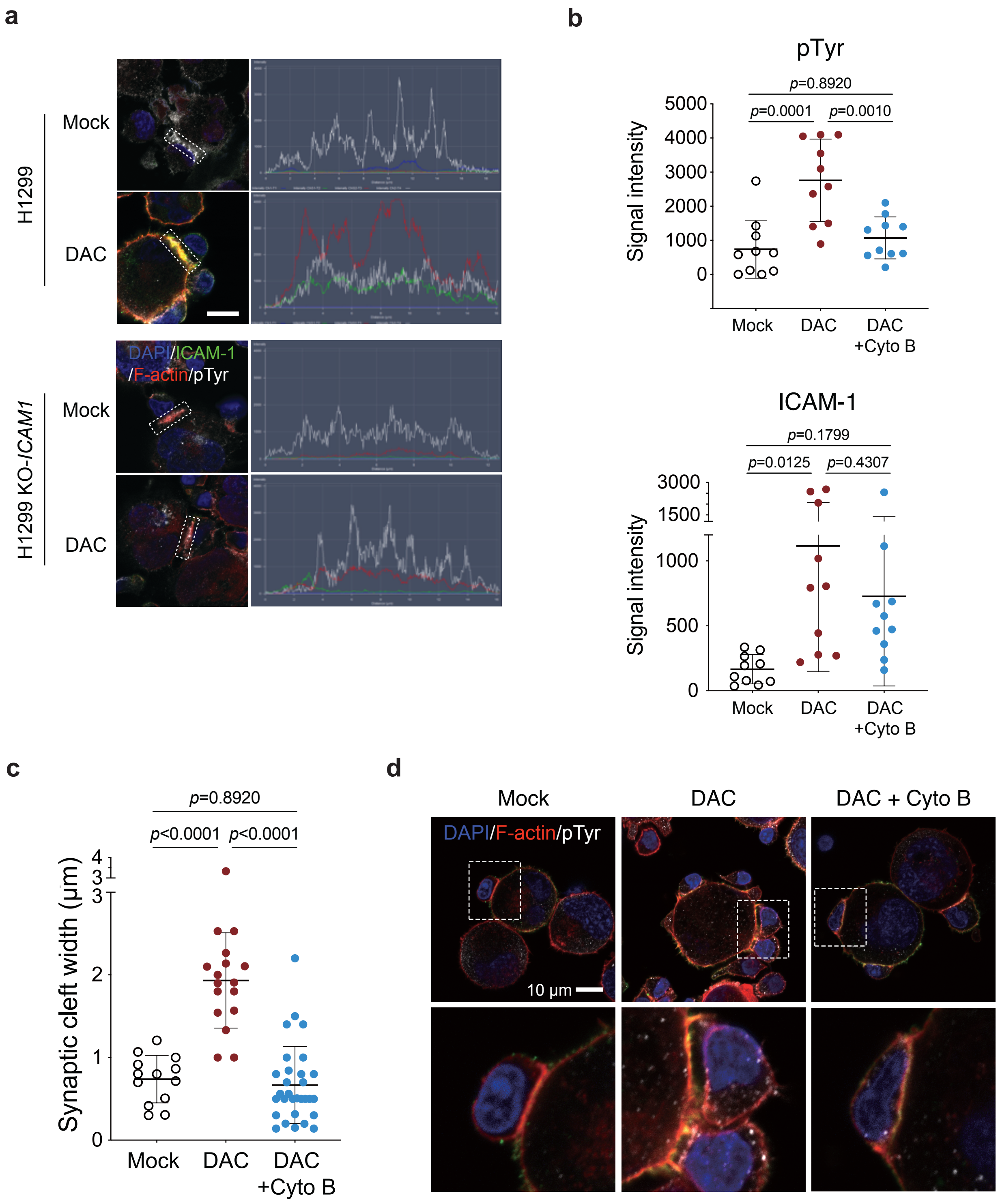

Supplementary Fig. 9 Inhibition of actin polymerization by cytochalasin B disrupts the decitabine-enhanced formation of active immune synapses. a Representative immunofluorescence staining of F-actin (red), ICAM-1 (green), and pTyr (phosphotyrosine,

white) at immune synapses between γδ T cells and H1299 lung cancer cells. Parental or *ICAM1* knockout (KO-*ICAM1*) H1299 cells are pretreated daily with PBS (Mock) or 100 nM decitabine (DAC) for 72 hours, followed by 3-day drug-free culture before coculture with γδ T cells. Signal intensities of each protein (F-actin, ICAM-1, pTyr) along the immune synapse area are graphed on the right. DAPI: 4′,6-diamidino-2-phenylindole, as a nuclear counterstain. Scale bar: 10 μm. Three independent experiments were performed. **b** Dot plots of pTyr and ICAM-1 signal intensities at immune synapses between γδ T cells and H1299 cells. H1299 cells are pretreated with PBS (Mock), DAC alone or a combination of DAC pretreatment (D3R3) and 1 μg/mL cytochalasin B (Cyto B) for 1.5 hours prior to coculture with γδ T cells (mean ± SD). *p* value is calculated by one-way ANOVA with Tukey's multiple comparisons test (n=10 over three independent experiments). **c** Dot plots showing the width of the immune synaptic cleft between γδ T cells and H1299 cells. H1299 cells are pretreated with PBS (Mock), DAC alone, or a combination of DAC and Cyto B. Data are presented as mean ± SD. *p* value is calculated by one-way ANOVA with Tukey's multiple comparisons test (Mock, n=13; DAC, n=17; DAC + Cyto B, n=30, over two independent experiments). **d** Representative immunofluorescence images of synaptic clefts stained for F-actin and pTyr. Scale bar: 10 μm. Three independent experiments were performed.

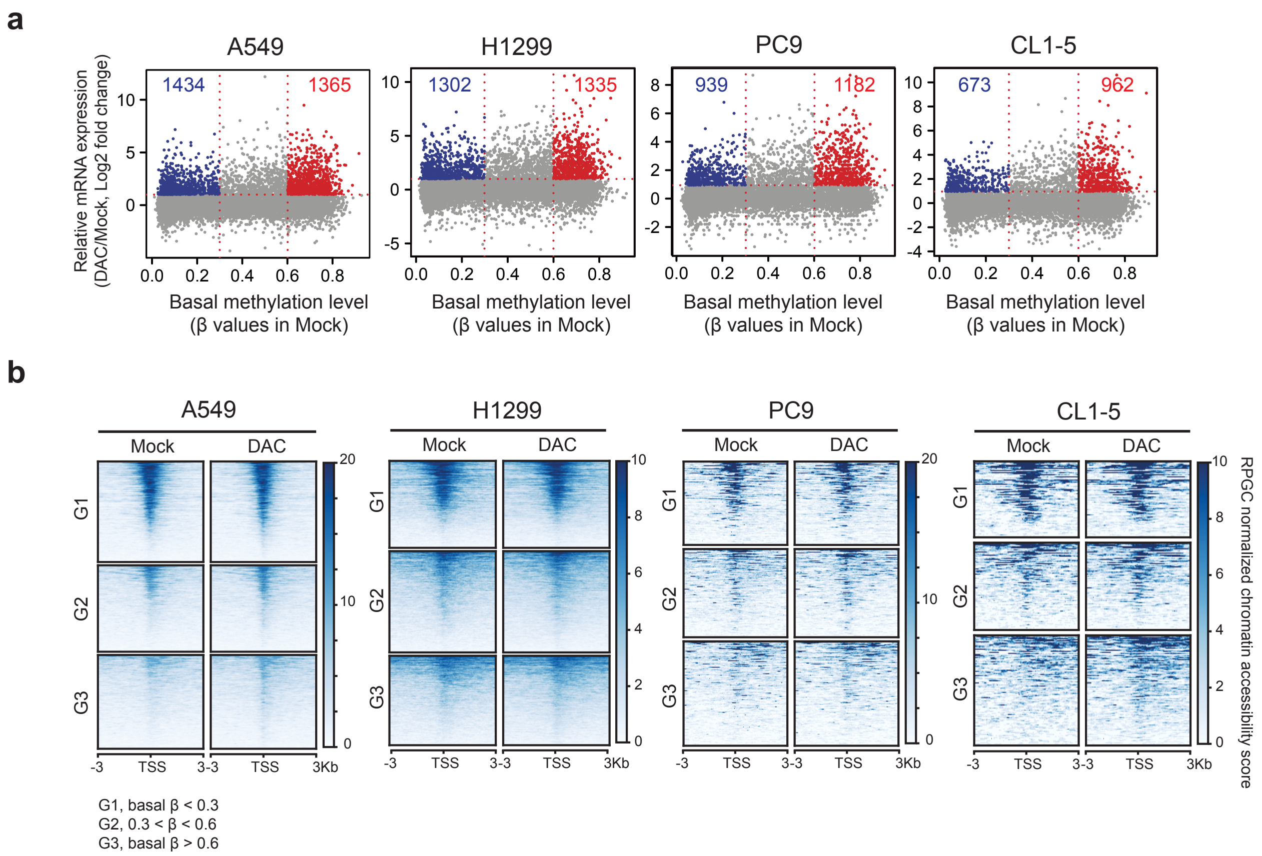

Supplementary Fig. 10 Integrative genome-wide analysis of DNA methylation and chromatin accessibility in lung cancer cells after decitabine treatment. a Transcriptional changes (y-axis, log2 fold change) in A549, H1299, PC9, CL1-5 cells following decitabine (DAC) treatment at D3R3 and basal methylation levels (x-axis) in cells without DAC treatment (Mock) for all genes measured by mRNA-seq and Infinium MethylationEPIC arrays, respectively. Increases of gene expression by at least 2-fold (Log2 fold change ≥ 1) are considered upregulated by DAC. The methylation levels represent the median β values of promoter probes for each gene. β value = 1, completely methylated; β value = 0, completely unmethylated. **b** Promoter chromatin accessibility measured by Omni-ATAC-seq for genes upregulated at least two-fold by DAC in H1299, PC-9, and CL1-5 lung cancer cells. Genes are classified into 3 groups based on β values at baseline (Mock) — G1 (β < 0.3), G2 (0.3 < β <0.6)

and G3 (β > 0.6). Chromatin accessibility around transcription start sites (TSS, -3 to +3 kb) is graphed. RPGC = reads per genomic content.

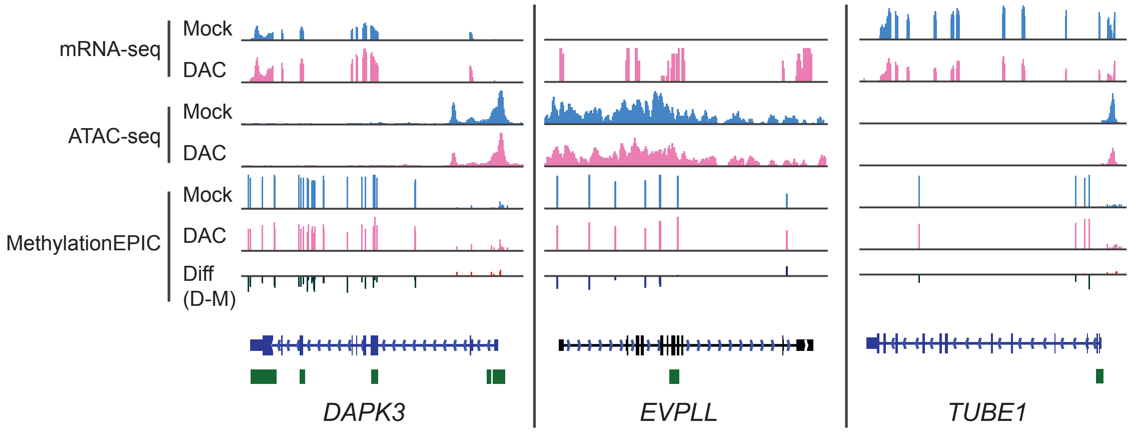

Supplementary Fig. 11 Visualization of multi-omics data in H1299 lung cancer cells following decitabine treatment. Three genes involved in each of the cytoskeletal modules (*DAPK3*, actin module; *EVPLL*, intermediate filament module; *TUBE1*, microtubule module) are used to exemplify the coordinated regulation between decitabine (DAC)-induced epigenetic alternations and mRNA expression levels by multi-omics data (i.e., mRNA-seq, Omni-ATAC-seq, and MethylationEPIC arrays). Diff (D–M): The difference of β values between DAC-treated and Mock-treated cells.

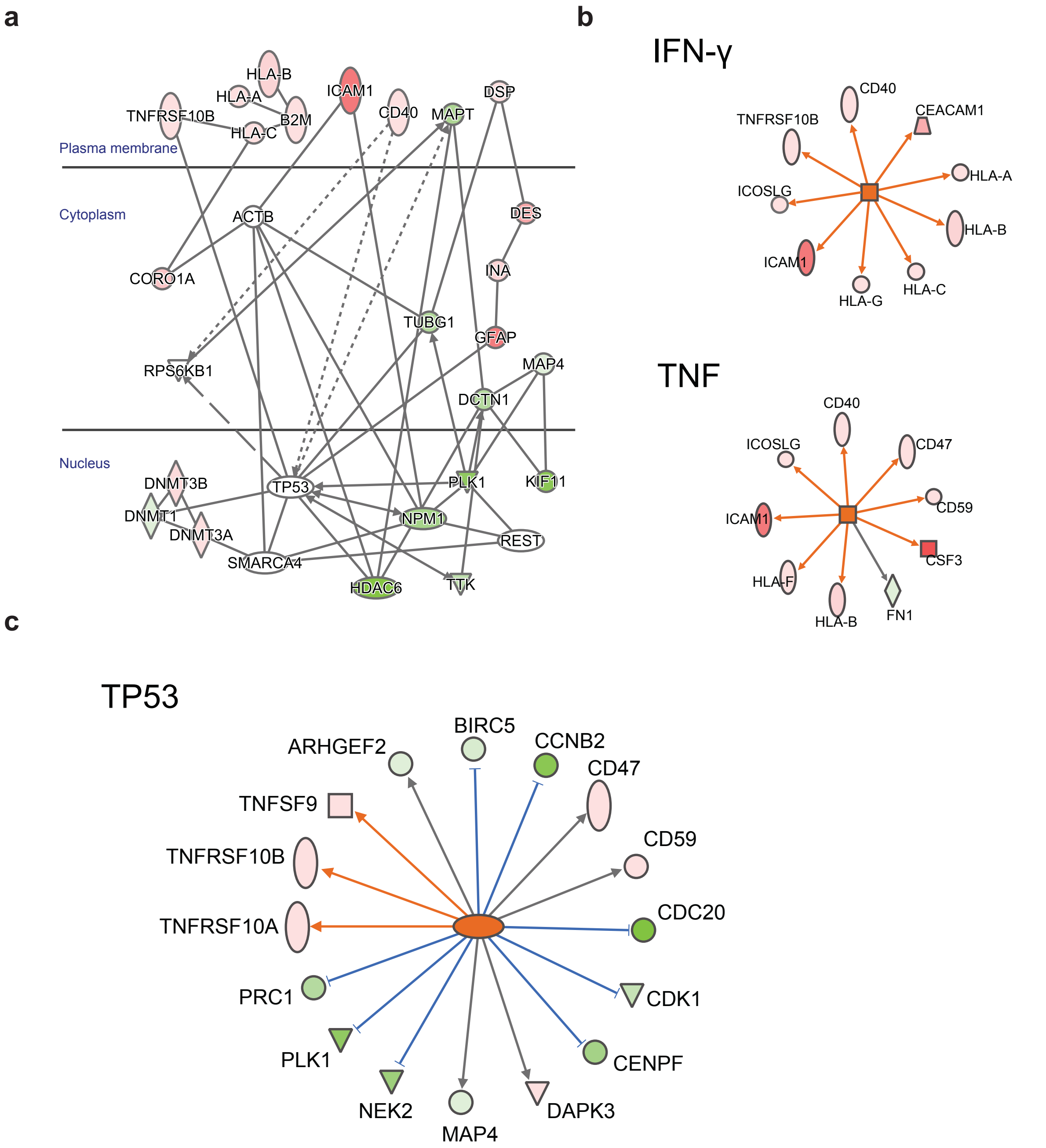

Supplementary Fig. 12 Network analysis reveals TP53 as a hub for interactions of immune synaptic molecules, the cytoskeleton, and epigenetic proteins. a IPA Network analysis of mRNA expression changes in human lung cancer cells treated by DAC reveals coordinated changes of the immune-related surface molecules and the cytoskeleton-associated genes. **b**, **c** IPA upstream regulator analysis of mRNA expression changes in human lung cancer cells

treated by DAC. **b** T cell effector cytokines such as TNF and IFN-γ may enhance DAC-induced expression changes of immune-related molecules and ICAM-1 in lung cancer cells. **c** TP53 is a potential master regulator for cancer cytoskeleton reorganization essential for DAC-potentiated γδ T cell killing.

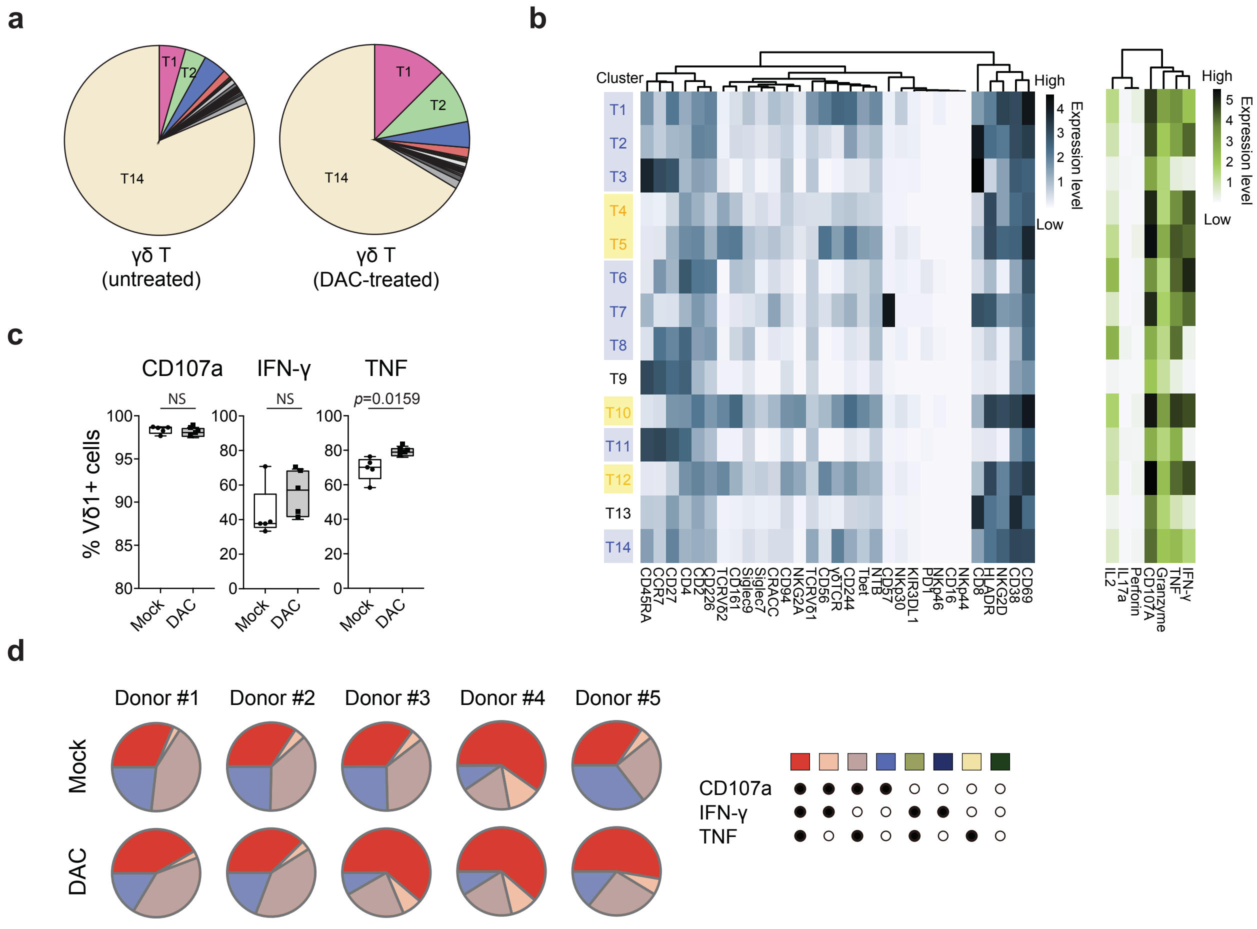

Supplementary Fig. 13 Single-cell mass cytometry characterizes functional γδ T cell subsets modulated by decitabine. a Pie charts showing the cell frequencies of 14 cell clusters (T1 to T14) revealed by clustering analysis of *ex vivo* expanded γδ T cells with or without decitabine (DAC) treatment based on 38 mass cytometric markers. **b** Heatmaps showing the mean intensity of each marker in the 14 cell clusters (T1 to T14). Dark blue and dark green are high, and white is low. The clusters belonging to Vδ1 or Vδ2 are labeled in blue and yellow, respectively. **c** Flow cytometric analysis of effector cytokine production by *ex vivo* expanded γδ T cells from 5 healthy donors at D3R3 with 10 nM DAC or mock treatment. Data are presented in box and whisker plots showing the percentages of Vδ1+ γδ T cells expressing individual cytokines. The box denotes the 25th percentile, the median, and the 75th percentile. The whiskers indicate minimum and maximum values. The *p* value is calculated by the two-sided Mann-Whitney test.

d Pie charts showing percentages of monofunctional and polyfunctional *ex vivo* expanded Vδ1+ γδ T cells from 5 healthy donors at D3R3 with 10 nM DAC or mock treatment. Percentages of Vδ1+ γδ T with a concurrent expression of three effector cytokines are marked in red.

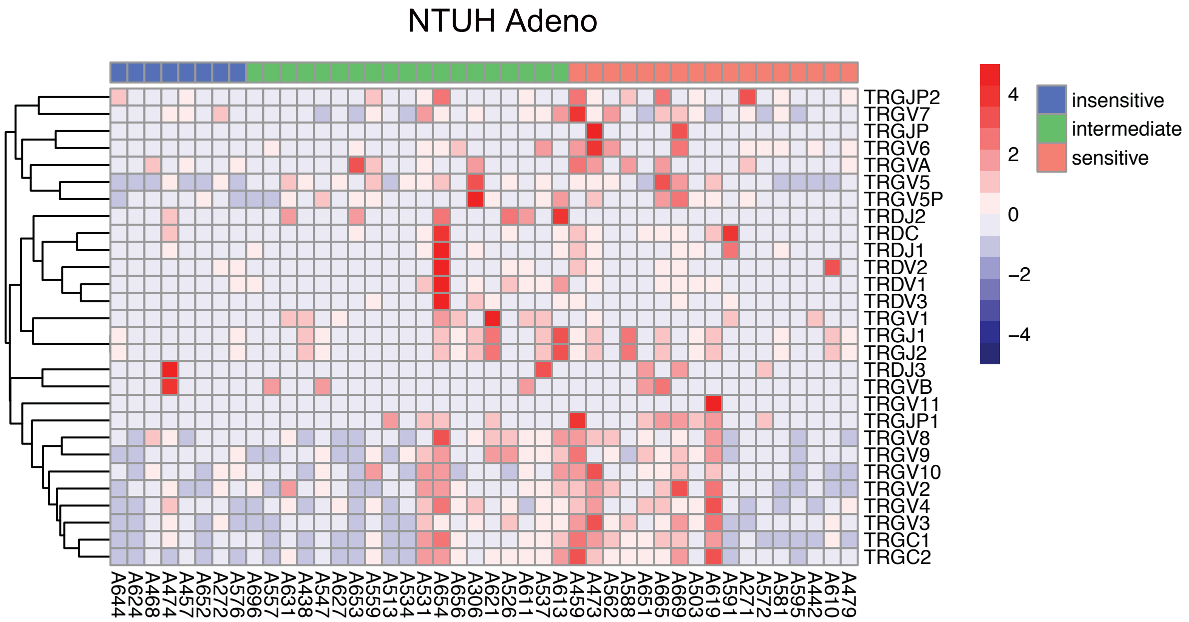

Supplementary Fig. 14 Detection of γδ TCR gene expressions in primary lung adenocarcinoma tissues. A heatmap of γδ TCR-related genes (i.e., gamma chain and delta chain) derived from mRNA-seq data of primary lung adenocarcinoma tumor tissues in patients at National Taiwan University Hospital (NTUH). The data show that higher levels of γδ TCR gene expression are detected in the immune-sensitive/immune-intermediate groups as compared with low γδ TCR gene expression in the immune-insensitive group.

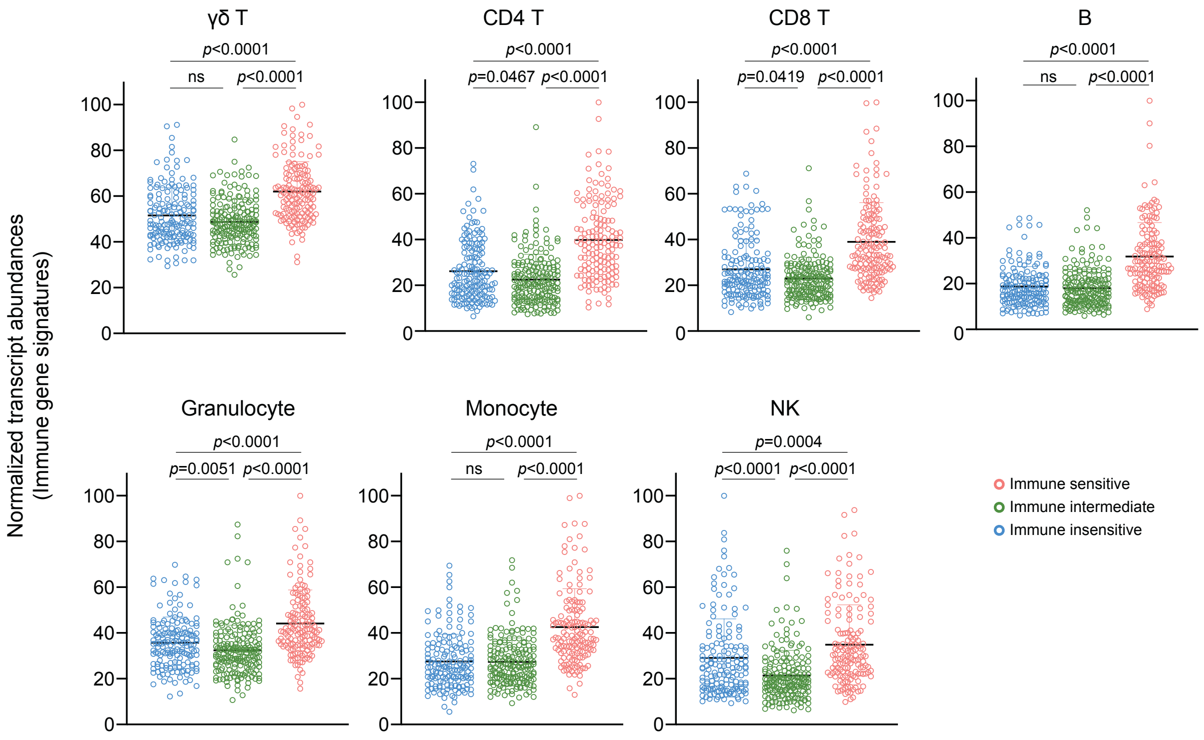

Supplementary Fig. 15 Deconvolution of transcriptomic data reveals relative abundances of infiltrating leukocytes in TCGA primary lung cancer tissues. Primary lung cancer tissues from TCGA were stratified into three groups— immune-sensitive, immune-intermediate, and immune-insensitive— based on the cytoskeletal gene signature. Gene signatures specific for individual immune cell types were based on CIBERSORT-LM7 reference gene signature matrix, derived from deconvolution of bulk tumor transcriptomes using a modified CIBERSORT algorithm by Tosolini *et al*. The *p* value was calculated by one-way ANOVA (immune-sensitive, n=149; immune-intermediate, n=190; immune-sensitive n=160 tissue samples; mean ± SD). ns, non-significant.

a

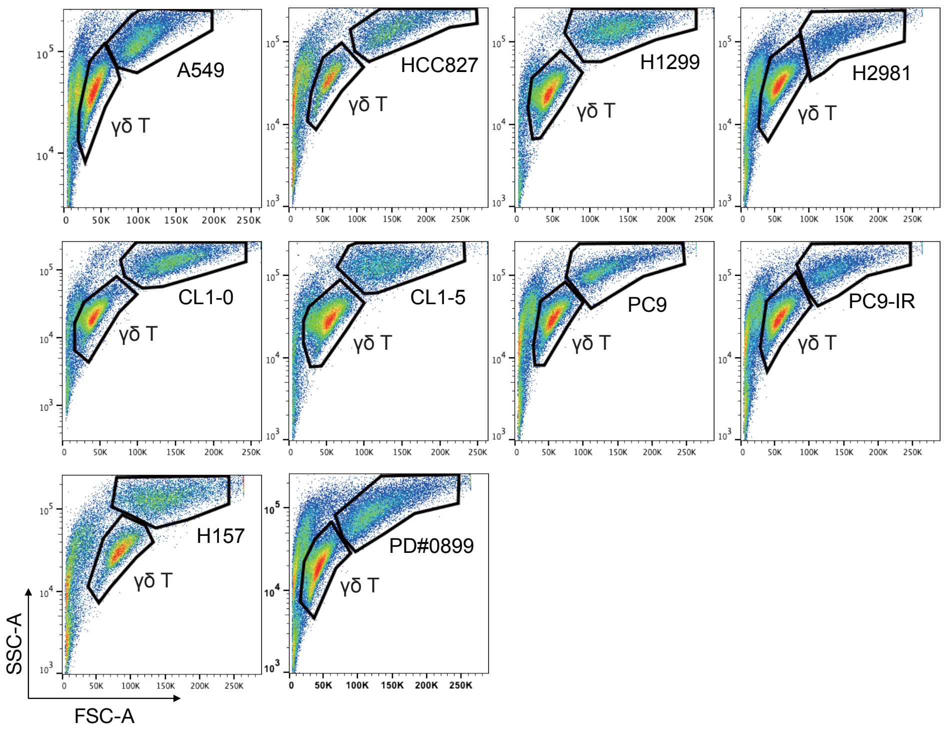

b

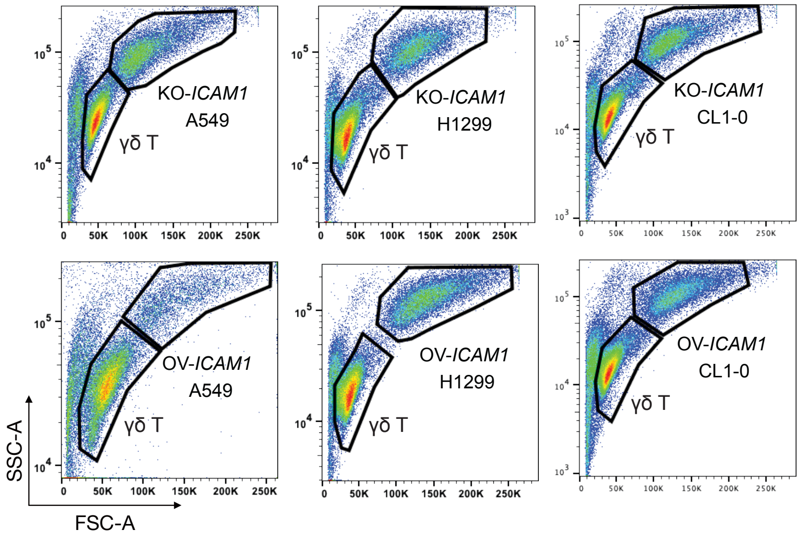

Supplementary Fig. 16 Gating strategies for flow cytometric analysis. a Gating strategies for Fig. 2d. **b** Gating strategies for Figures 3e, g and Supplementary figs 6b. FSC: forward scatter. SSC: side scatter.

Supplementary Tables

Supplementary Table 1. List of cell lines

Cell Lines	Source	Identifier
Human: 293T (Embryonic kidney)	ATCC	ATCC CRL-3216
Human: A549 (Lung adenocarcinoma)	ATCC	ATCC CCL-185; Authenticated by STR analysis
Human: CL1-0 (Lung adenocarcinoma)	National Taiwan University	Authenticated by STR analysis
Human: CL1-5 (Lung adenocarcinoma)	National Taiwan University	Authenticated by STR analysis
Human: H157 (Lung squamous carcinoma)	ATCC	ATCC CCL-5802; Authenticated by STR analysis
Human: H1299 (Lung adenocarcinoma)	ATCC	ATCC CCL-5803; Authenticated by STR analysis
Human: H1792 (Lung adenocarcinoma)	ATCC	ATCC CRL-5895
Human: H2170 (Lung squamous carcinoma)	ATCC	ATCC CCL-5928; Authenticated by STR analysis
Human: H2981 (Lung adenocarcinoma)	ATCC	ATCC CCL-5803
Human: HCC827 (Lung adenocarcinoma)	ATCC	ATCC CCL-2868; Authenticated by STR analysis
Human: HCT116 (Colorectal carcinoma)	ATCC	ATCC CCL-247
Human: PC9 (Lung adenocarcinoma)	National Taiwan University	Authenticated by STR analysis
Human: PC9/IR (Lung adenocarcinoma)	National Taiwan University	Authenticated by STR analysis

Supplementary Table 2. List of antibodies

Antibodies	Source	Dilution or Conc. (ug/mL)	Identifier
ß-Actin; Mouse; Clone C4; N/A; WB	Millipore	1:1000 – 1:5000	Cat# MAB1501; RRID:AB_2223041
CD2; Mouse; Clone RPA-2.10; 147Sm; CyTOF	Biolegend	2	Cat# 300202; RRID:AB_314026
CD3; Mouse; Clone BW264/56; APC; FC	Miltenyi Biotec	1:50	Cat# 130-113-125; RRID:AB_2725953
CD3; Mouse; Clone UCHT1; 113In; CyTOF	Biolegend	2	Cat# 300402; RRID:AB_314056
CD3; Mouse; Clone UCHT1; N/A; IF	BioLegend	1:300	Cat# 300416; RRID:AB_389332
CD4; Rat; Clone A161A1; 115In; CyTOF	Biolegend	2	Cat# 357402; RRID:AB_2561907
CD8a; Mouse; Clone RPA-T8; 146Nd; CyTOF	Fluidigm	1:100	Cat# 3146001B; RRID:AB_2687641
CD11a (LFA-1); Mouse; Clone HI111; N/A; IF	BioLegend	1:500	Cat# 301202; RRID:AB_314140
CD14; Mouse; Clone M5E2; 173Yb; CyTOF	Biolegend	1	Cat# 301802; RRID:AB_314184
CD16; Mouse; Clone 3G8; 165Ho; CyTOF	Biolegend	1	Cat# 302002; RRID:AB_314202
CD16; Mouse; Clone 3G8; Brilliant Violet 421; FC	Sony	1:50	Cat# 2110190; RRID:N/A
CD19; Mouse; Clone HIB19; 173Yb; CyTOF	Biolegend	1	Cat# 302202; RRID:AB_314232
CD27; N/A; Clone L128; 167Er; CyTOF	BD Biosciences	2	Cat# Custom-order; RRID:N/A
CD38; Mouse; Clone HB-7; 145Nd; CyTOF	Biolegend	2	Cat# 356602; RRID:AB_2561794
CD45; N/A; Clone HI30; 89Y; CyTOF	Fluidigm	1:400	Cat# 3089003B; RRID:AB_2661851
CD45RA; Mouse; Clone HI100; 143Nd; CyTOF	Biolegend	2	Cat# 304102; RRID:AB_314406
CD56; Mouse; Clone NCAM16.2; Brilliant Violet 421; FC	BD Biosciences	1:50	Cat# 562751; RRID:AB_2732054
CD56; Mouse; Clone NCAM16.2; 176Yb; CyTOF	Fluidigm	1:100	Cat# 3176008B; RRID:AB_2661813
CD57; Mouse; Clone HNK-1; 139La; CyTOF	Biolegend	0.2	Cat# 359602; RRID:AB_2562403

CD66b; Mouse; Clone G10F5; 141Pr; CyTOF	Biolegend	2	Cat# 305102; RRID:AB_314494
CD69; Mouse; Clone FN50; 162Dy; CyTOF	Biolegend	2	Cat# 310902; RRID:AB_314837
CD69; Mouse; Clone FN50; PerCP/Cyanine5.5; FC	BioLegend	1:50	Cat# 310925; RRID:AB_2074957
CD94; Mouse; Clone DX22; 144Nd; CyTOF	Biolegend	2	Cat# 305502; RRID:AB_314532
CD107a; Mouse; Clone H4A3; 151Eu; CyTOF	BD Biosciences	2	Cat# 555798; RRID:AB_396132
CD107a; Mouse; Clone H4A3; Brilliant Violet 605; FC	BioLegend	1:50	Cat# 328634; RRID:AB_2563851
CD107a; Mouse; Clone H4A3; Phycoerythrin; FC	BD Pharmingen	1:50	Cat# 555801; RRID:AB_396135
CD107b; Mouse; Clone H4B4; 151Eu; CyTOF	BD Biosciences	2	Cat# 555803; RRID:AB_396137
CD159a (NKG2A); Mouse; Clone Z199; APC; 170Er; CyTOF	Beckman Coulter	2	Cat# A60797; RRID:AB_10643105
CD161; Mouse; Clone HP-3G10; 164Dy; CyTOF	Biolegend	2	Cat# 339902; RRID:AB_1501090
CD197 (CCR7); Mouse; Clone G043H7; 159Tb; CyTOF	Fluidigm	1:100	Cat# 3159003A; RRID:AB_2714155
CD226 (DNAM-1); Mouse; Clone TX25; 149Sm; CyTOF	Biolegend	2	Cat# 337102; RRID:AB_1236383
CD235ab; Mouse; Clone HIR2; 141Pr; CyTOF	Biolegend	2	Cat# 306602; RRID:AB_314620
CD244 (2B4); Mouse; Clone C1.7; 163Dy; CyTOF	Biolegend	2	Cat# 329502; RRID:AB_1279194
CD279 (PD-1); Mouse; Clone EH12.2H7; 174Yb; CyTOF	Fluidigm	1:100	Cat# 3174020B; RRID:AB_2687629
CD314 (NKG2D); Mouse; Clone ON72; 166Er; CyTOF	Fluidigm	1:100	Cat# 3166016B; RRID:AB_2801262
CD319 (CRACC); Mouse; Clone 162.1; 155Gd; CyTOF	Biolegend	0.2	Cat# 331802; RRID:AB_961330
CD328 (Siglec-7); Mouse; Clone 6-434; 153Eu; CyTOF	Biolegend	2	Cat# 339202; RRID:AB_1501162
CD329 (Siglec-9); Mouse; Clone K8; 158Gd; CyTOF	Biolegend	2	Cat# 351502; RRID:AB_10896430
CD335 (NKp46); Mouse; Clone 195314; 160Gd; CyTOF	R&D Systems	2	Cat# MAB1850-100; RRID:AB_2149153
CD336 (NKp44); Mouse; Clone P44-8; 169Tm; CyTOF	Biolegend	1	Cat# 325102; RRID:AB_756094
CD337 (NKp30); Mouse; Clone P30-15; 172Yb; CyTOF	Biolegend	2	Cat# 325202; RRID:AB_756106

CD352 (NTB-A); Mouse; Clone NT-7; 154Sm; CyTOF	Biolegend	4	Cat# 317202; RRID:AB_571931
EpCAM; Mouse; Clone EBA-1; PE; FC	BD Biosciences	1:50	Cat#347198; RRID: AB_400262
Granzyme B; Mouse; Clone GB11; 171Yb; CyTOF	Fluidigm	1:200	Cat# 3171002B; RRID:AB_2687652
HLA-DR; Mouse; Clone L243; 140Ce; CyTOF	Biolegend	2	Cat# 307602; RRID:AB_314680
ICAM-1; Mouse; Clone BBIG-I1; FITC; FC	R&D Systems	1:50	Cat# BBA20; RRID:AB_356942
ICAM-1; Rabbit; Clone EPR4776; N/A; IF	Abcam	1:250	Cat# ab109361; RRID:AB_10858467
ICAM-1; Rabbit; N/A; N/A; WB	Cell Signaling Technology	1:1000	Cat# 4915; RRID:AB_2280018
IFN-γ; Mouse; Clone 25723.11; Allophcocyanin; FC	BD Biosciences	1:50	Cat# 341117; RRID:AB_2264629
IFN-γ; Mouse; Clone B27; 209Bi; CyTOF	BD Biosciences	1	Cat# 554698; RRID:AB_395516
IL-2; Rat; Clone MQ1-17H12; 157Gd; CyTOF	Biolegend	2	Cat# 500302; RRID:AB_315089
IL-10; Rat; Clone JES3-19F1; APC; FC	BioLegend	1:50	Cat# 506806; RRID:AB_315456
IL-17A; Mouse; Clone BL168; 168Er; CyTOF	Biolegend	1	Cat# 512302; RRID:AB_961399
IL-17A; Mouse; Clone BL168; Brilliant Violet 421; FC	Biolegend	1:50	Cat# 512321; RRID:AB_10899566
KIR3DL1; Mouse; Clone DX9; 148Nd; CyTOF	R&D Systems	2	Cat# MAB1225; RRID:AB_2130828
LAT; Mouse; N/A; N/A; IF	Santa Cruz	1:200	Cat# sc-53550; RRID:AB_784283
Perforin; Mouse; Clone B-D48; 175Yb; CyTOF	Fluidigm	1:400	Cat# 3175004B; RRID:N/A
Phosphotyrosin; Mouse; Clone 4G10; N/A; IF	Merck Millipore	1:200	Cat# 05-321; RRID:AB_568857
T-bet; Mouse; Clone 4B10; 161Dy; CyTOF	Biolegend	2	Cat# 644802; RRID:AB_1595503
TCR γδ; Mouse; Clone B1; 152Sm; CyTOF	Biolegend	6	Cat# 331202; RRID:AB_1089222
TCR γδ; Mouse; Clone GL3; FITC; FC	Miltenyi Biotec	1:50	Cat# 130-104-015; RRID:AB_2654076
TCR PAN γδ; Mouse; Clone IMMU510; N/A; FC	Beckman Coulter	1:50	Cat# IM1349; RRID:AB_131619
TCR Vδ1; Mouse; Clone REA173; FITC; FC, CyTOF	Miltenyi Biotec	1:200	Cat# 130-118-498; RRID:AB_2751531

TCR Vδ2; Mouse; Clone B6; 142Nd; CyTOF	Biolegend	1	Cat# 331402; RRID:AB_1089226
TCR Vδ2; Mouse; Clone B6; Brilliant Violet 711; FC	BioLegend	1:50	Cat# 331412; RRID:AB_2565421
TNF; Mouse; Clone MAB11; 156Gd; CyTOF	Biolegend	0.5	Cat#502902; RRID:AB_315254
TNF; Mouse; Clone MAB11; PE-Cy7; FC	BD Biosciences	1:50	Cat# 560678; RRID:AB_1727578
Anti-Mouse IgG (H+L) Cross-Adsorbed Secondary Antibody, Alexa Fluor 488; Goat; IF	Thermo Fisher Scientific	1:200	Cat# A-11001; RRID:AB_2534069
Anti-Mouse IgG (H+L) Cross-Adsorbed Secondary Antibody, Alexa Fluor 647; Goat; IF	Thermo Fisher Scientific	1:200	Cat# A-21235; RRID:AB_2535804
Anti-Mouse IgG; Goat; Clone Poly4053; HRP; WB	BioLegend	1:2000 – 1:4000	Cat# 405306; RRID:AB_315009
Anti-Rabbit IgG (H+L) Cross-Adsorbed Secondary Antibody, Alexa Fluor 488; Goat; IF	Thermo Fisher Scientific	1:200	Cat# A-11008; RRID:AB_143165
Anti-Rabbit IgG (H+L) Cross-Adsorbed Secondary Antibody, Alexa Fluor 647; Goat; IF	Thermo Fisher Scientific	1:200	Cat# A-21244; RRID:AB_2535812
Anti-Rabbit IgG; Goat; N/A; HRP; WB	GeneTex	1:2000 – 1:4000	Cat# GTX213110-01; RRID:AB_10618573
Fluorescein/Oregon Green; Rabbit; 150Nd; FITC; CyTOF	Thermo Fisher Scientific	0.5	Cat# A-889; RRID:AB_221561

*Human protein target; Host; Clone; Conjugation; For the analysis in this study (IF, immunofluorescence; CyTOF, mass cytometry; FC; flow cytometry; WB, Western blot)

Supplementary Table 3. List of antibody labeling kits for metal isotope conjugation

Miscellaneous	Source	Identifier
Maxpar® X8 Antibody Labeling Kit, 141Pr	Fluidigm	Cat# 201141
Maxpar® X8 Antibody Labeling Kit, 142Nd	Fluidigm	Cat# 201142
Maxpar® X8 Antibody Labeling Kit, 143Nd	Fluidigm	Cat# 201143
Maxpar® X8 Antibody Labeling Kit, 144Nd	Fluidigm	Cat# 201144
Maxpar® X8 Antibody Labeling Kit, 145Nd	Fluidigm	Cat# 201145
Maxpar® X8 Antibody Labeling Kit, 146Nd	Fluidigm	Cat# 201146
Maxpar® X8 Antibody Labeling Kit, 147Sm	Fluidigm	Cat# 201147
Maxpar® X8 Antibody Labeling Kit, 148Nd	Fluidigm	Cat# 201148
Maxpar® X8 Antibody Labeling Kit, 149Sm	Fluidigm	Cat# 201149
Maxpar® X8 Antibody Labeling Kit, 150Nd	Fluidigm	Cat# 201150
Maxpar® X8 Antibody Labeling Kit, 151Eu	Fluidigm	Cat# 201151
Maxpar® X8 Antibody Labeling Kit, 152Sm	Fluidigm	Cat# 201152
Maxpar® X8 Antibody Labeling Kit, 153Eu	Fluidigm	Cat# 201153
Maxpar® X8 Antibody Labeling Kit, 154Sm	Fluidigm	Cat# 201154
Maxpar® X8 Antibody Labeling Kit, 155Gd	Fluidigm	Cat# 201155
Maxpar® X8 Antibody Labeling Kit, 156Gd	Fluidigm	Cat# 201156
Maxpar® X8 Antibody Labeling Kit, 158Gd	Fluidigm	Cat# 201158
Maxpar® X8 Antibody Labeling Kit, 159Tb	Fluidigm	Cat# 201159
Maxpar® X8 Antibody Labeling Kit, 160Gd	Fluidigm	Cat# 201160
Maxpar® X8 Antibody Labeling Kit, 161Dy	Fluidigm	Cat# 201161
Maxpar® X8 Antibody Labeling Kit, 162Dy	Fluidigm	Cat# 201162
Maxpar® X8 Antibody Labeling Kit, 163Dy	Fluidigm	Cat# 201163
Maxpar® X8 Antibody Labeling Kit, 164Dy	Fluidigm	Cat# 201164
Maxpar® X8 Antibody Labeling Kit, 165Ho	Fluidigm	Cat# 201165
Maxpar® X8 Antibody Labeling Kit, 166Er	Fluidigm	Cat# 201166
Maxpar® X8 Antibody Labeling Kit, 167Er	Fluidigm	Cat# 201167
Maxpar® X8 Antibody Labeling Kit, 168Er	Fluidigm	Cat# 201168
Maxpar® X8 Antibody Labeling Kit, 169Tm	Fluidigm	Cat# 201169
Maxpar® X8 Antibody Labeling Kit, 170Er	Fluidigm	Cat# 201170
Maxpar® X8 Antibody Labeling Kit, 171Yb	Fluidigm	Cat# 201171
Maxpar® X8 Antibody Labeling Kit, 172Yb	Fluidigm	Cat# 201172
Maxpar® X8 Antibody Labeling Kit, 173Yb	Fluidigm	Cat# 201173
Maxpar® X8 Antibody Labeling Kit, 174Yb	Fluidigm	Cat# 201174
Maxpar® X8 Antibody Labeling Kit, 175Lu	Fluidigm	Cat# 201175
Maxpar® X8 Antibody Labeling Kit, 176Yb	Fluidigm	Cat# 201176

Supplementary Table 4. Primer sequences

Oligonucleotides	Source	Identifier
Forward primer for cloning full length *ICAM1* cDNA: CGGGATCCATGGCTCCCAGCAGCCC	This study	N/A
Reverse primer for cloning full length *ICAM1* cDNA: CGGGATCCTCAGGGAGGCGTGGCTT	This study	N/A
Forward primer for the *ICAM1* ATAC-PCR: ACCGTGATTCAAGCTTAGCC	This study	N/A
Reverse primer for the *ICAM1* ATAC-PCR: CCGGAACAAATGCTGCAGTT	This study	N/A
Forward primer for PCR amplification of the flanking regions surrounding the sgRNA sequence: TCCACATCGAAGGCAAAGTAT	This study	N/A
Reverse primer for PCR amplification of the flanking regions surrounding the sgRNA sequence: CCCCTCCTTGACCCTACGA	This study	N/A

Supplementary Table 5. List of software programs

Software	Source	Link
ATACseqQC (v1.8.5)	Ou, et al., 2018	https://bioconductor.org/packages/release/bioc/html/ATACseqQC.html
BWA (v0.7.17-r1188)	Li, et al., 2009	http://bio-bwa.sourceforge.net/
bowtie2 (v2.2.6)	Langmead, et al., 2012	http://bowtie-bio.sourceforge.net/bowtie2/index.shtml
Bioconductor (v3.9)	Bioconductor	https://www.bioconductor.org/
CutAdapt (v2.7)	Martin, 2011	https://journal.embnet.org/index.php/embnetjournal/article/view/200/479
curatedTCGAData (v1.6.0)	Ramos, 2019	http://bioconductor.org/packages/release/data/experiment/html/curatedTCGAData.html
ChIPseeker (v1.20.0)	Yu, et al., 2015	https://bioconductor.org/packages/release/bioc/html/ChIPseeker.html
Cytobank	Cytobank, Inc.	http://www.cytobank.org/)
deepTools (v3.3.1)	Ramírez, et al., 2016	https://deeptools.readthedocs.io/en/develop/
DESeq2 (v1.24.0)	Love, et al., 2014	https://bioconductor.org/packages/release/bioc/html/DESeq2.html
FlowJo (v10)	FlowJo	https://www.flowjo.com/
ggplot2 (v3.2.1)	Wickham, 2016	https://cran.r-project.org/web/packages/ggplot2/index.html
GenomicFeatures (v1.36.4)	Lawrence, et al., 2013	https://bioconductor.org/packages/release/bioc/html/GenomicFeatures.html
GenomicAlignments (1.20.1)	Lawrence, et al., 2013	https://bioconductor.org/packages/release/bioc/html/GenomicAlignments.html
GraphPad Prism 8	GraphPad	https://www.graphpad.com/
IGV (v2.5.0)	James, et al., 2011	https://software.broadinstitute.org/software/igv/
IlluminaHumanMethylationEPICmanifest (v0.3.0)	Hansen, 2016	https://bioconductor.org/packages/release/data/annotation/html/IlluminaHumanMethylationEPICmanifest.html
IlluminaHumanMethylationEPICanno.ilm10b4.hg19 (v0.6.0)	Hansen, 2017	https://bioconductor.org/packages/release/data/annotation/html/IlluminaHumanMethylationEPICanno.ilm10b4.hg19.html
ImageJ (v2.0.0-rc-69/1.52p)	ImageJ	https://imagej.nih.gov/ij/
javaGSEA (v2.2.4)	Tamayo, et al., 2005	http://software.broadinstitute.org/gsea/index.jsp

limma (v3.40.6)	Ritchie, et al., 2015	https://bioconductor.org/packages/release/bioc/html/limma.html
minfi (v1.30.0)	Aryee, et al., 2014	https://bioconductor.org/packages/release/bioc/html/minfi.html
MACS2 (v2.2.5)	Zhang, et al., 2008	https://github.com/taoliu/MACS
MaxQuant (v1.6.0.16)	Cox, et al., 2008	https://www.biochem.mpg.de/5111795/maxquant
pheatmap (v1.0.12)	Kolde, 2019	https://cran.r-project.org/web/packages/pheatmap/index.html
CLC Genomics Workbench	QIAGEN	https://digitalinsights.qiagen.com/products-overview/discovery-insights-portfolio/analysis-and-visualization/qiagen-clc-genomics-workbench/
R-Studio for macOS (v1.2.1335)	R-Studio	https://www.rstudio.com/
R-base (v 3.6.1)	The R foundation	https://www.r-project.org/
RSEM (v1.2.23)	Bo and Colin, 2011	https://github.com/deweylab/RSEM
SAMtools (v1.9)	Li, et al., 2009	http://samtools.sourceforge.net/
survminer (v0.4.6)	Kassambara, et al., 2019	https://cran.r-project.org/web/packages/survminer/index.html
survival (v3.1-8)	Therneau, 2015	https://cran.r-project.org/web/packages/survminer/index.html
Trimmomatic (v0.33)	Bolger, et al., 2014	http://www.usadellab.org/cms/index.php?page=trimmomatic
Vortex (26-Apr-2018)	Samusik, et al., 2016	https://web.stanford.edu/~samusik/vortex/
Xcalibur (v3.0.63)	Thermo Fisher Scientific	https://www.thermofisher.com/order/catalog/product/OPTON-30487
ZEN (v14.0.12.201)	ZEISS	https://www.zeiss.com/microscopy/int/products/microscope-software/zen-lite.html
Ingenuity Pathway Analysis (IPA®, Version 01-16)	QIAGEN	https://digitalinsights.qiagen.com/products-overview/discovery-insights-portfolio/analysis-and-visualization/qiagen-ipa/
