# Supplementary material for: Epigenetic modulation of immune synaptic-cytoskeletal networks potentiates γδ T cell-mediated cytotoxicity in lung cancer
Source: Nat Commun. 2021 Apr 12;12:2163. doi: 10.1038/s41467-021-22433-4 (PMC8042060; doi:10.1038/s41467-021-22433-4)
Supplement: Supplementary file 3 — Reporting Summary [file 41467_2021_22433_MOESM3_ESM.pdf]

nature research

Corresponding author(s): Hsing-Chen Tsai, M.D., Ph.D.

Last updated by author(s): Feb 20, 2021

Reporting Summary

Nature Research wishes to improve the reproducibility of the work that we publish. This form provides structure for consistency and transparency in reporting. For further information on Nature Research policies, see our Editorial Policies and the Editorial Policy Checklist.

Statistics

For all statistical analyses, confirm that the following items are present in the figure legend, table legend, main text, or Methods section.

n/a	Confirmed	
☐	☒	The exact sample size (*n*) for each experimental group/condition, given as a discrete number and unit of measurement
☐	☒	A statement on whether measurements were taken from distinct samples or whether the same sample was measured repeatedly
☐	☒	The statistical test(s) used AND whether they are one- or two-sided<br>*Only common tests should be described solely by name; describe more complex techniques in the Methods section.*
☒	☐	A description of all covariates tested
☐	☒	A description of any assumptions or corrections, such as tests of normality and adjustment for multiple comparisons
☐	☒	A full description of the statistical parameters including central tendency (e.g. means) or other basic estimates (e.g. regression coefficient) AND variation (e.g. standard deviation) or associated estimates of uncertainty (e.g. confidence intervals)
☐	☒	For null hypothesis testing, the test statistic (e.g. *F*, *t*, *r*) with confidence intervals, effect sizes, degrees of freedom and *P* value noted<br>*Give P values as exact values whenever suitable.*
☒	☐	For Bayesian analysis, information on the choice of priors and Markov chain Monte Carlo settings
☒	☐	For hierarchical and complex designs, identification of the appropriate level for tests and full reporting of outcomes
☒	☐	Estimates of effect sizes (e.g. Cohen's *d*, Pearson's *r*), indicating how they were calculated

Our web collection on statistics for biologists contains articles on many of the points above.

Software and code

Policy information about availability of computer code

Data collection	ATACseqQC (v1.8.5); BWA (v0.7.17-r1188); bowtie2 (v2.2.6); Bioconductor (v3.9); CutAdapt (v2.7); curatedTCGAData (v1.6.0); ChIPseeker (v1.20.0); Cytobank; deepTools (v3.3.1); DESeq2 (v1.24.0); FlowJo (v10); ggplot2 (v3.2.1); GenomicFeatures (v1.36.4); GenomicAlignments (1.20.1); GraphPad Prism 8; IGV (v2.5.0); IlluminaHumanMethylationEPICmanifest (v0.3.0); IlluminaHumanMethylationEPICanno.ilm10b4.hg19 (v0.6.0); ImageJ (v2.0.0-rc-69/1.52p); javaGSEA (v2.2.4); limma (v3.40.6); minfi (v1.30.0); MACS2 (v2.2.5); MaxQuant (v1.6.0.16); pheatmap (v1.0.12); CLC Genomics Workbench; R-Studio for macOS (v1.2.1335); R-base (v 3.6.1); RSEM (v1.2.23); SAMtools (v1.9); survminer (v0.4.6); survival (v3.1-8); Trimmomatic (v0.33); Vortex (26-Apr-2018); Xcalibur (v3.0.63); ZEN (v14.0.12.201); Ingenuity Pathway Analysis (IPA®, Version 01-16)
Data analysis	No custom algorithms were used in this study.

For manuscripts utilizing custom algorithms or software that are central to the research but not yet described in published literature, software must be made available to editors and reviewers. We strongly encourage code deposition in a community repository (e.g. GitHub). See the Nature Research guidelines for submitting code & software for further information.

Data

Policy information about availability of data

All manuscripts must include a data availability statement. This statement should provide the following information, where applicable:

- Accession codes, unique identifiers, or web links for publicly available datasets
- A list of figures that have associated raw data
- A description of any restrictions on data availability

Genomic data generated in this study are available in the Gene Expression Omnibus (GEO) database under the accession numbers: “GSE145588 (genome-wide methylation) [https://www.ncbi.nlm.nih.gov/geo/query/acc.cgi?acc=GSE145588]”, “GSE145663 (mRNA-seq for lung cancer cell lines) [https://

www.ncbi.nlm.nih.gov/geo/query/acc.cgi?acc=GSE145663]", "GSE120622 (mRNA-seq data for lung cancer tissues) [https://www.ncbi.nlm.nih.gov/geo/query/acc.cgi?acc=GSE120622]" and "GSE145663 (Omni-ATAC-seq)[https://www.ncbi.nlm.nih.gov/geo/query/acc.cgi?acc=GSE145663]". The mRNA-seq data of colorectal cancer cell lines with DNMT depletion can be accessed at "GSE93136 [https://www.ncbi.nlm.nih.gov/geo/query/acc.cgi?acc=GSE93136]". The mRNA-seq data of human lung cancer tissues in the NTUH cohort can be accessed at "GSE120622 [https://www.ncbi.nlm.nih.gov/geo/query/acc.cgi?acc=GSE120622]". The CyTOF raw FCS files have been deposited to FlowRepository database with the identifier "FR-FCM-Z2G5 [http://flowrepository.org/id/RvFrCnAerbp0HtQDRL4RsHi6hlFBBoV8GLbAB4qsnHQdKuisCQZPJ4MIyqLMovBF]". The Thermo RAW files and MaxQuant results have been deposited to the ProteomeXchange Consortium with the dataset identifier "MSV000084997 [https://massive.ucsd.edu/ProteoSAFe/dataset.jsp?task=94de9653c2e74b4e9e9ba4a3db30b039]" through the MassIVE partner repository. Human reference proteome can be accessed at "UniprotKB [ftp://ftp.uniprot.org/pub/databases/uniprot/current_release/knowledgebase/reference_proteomes/Eukaryota/]". Full microscopy image data sets have been deposited to Mendeley Data (DOI: 10.17632/cx2mxszth9.1). Other source data supporting the findings of this study are provided with this paper.

Field-specific reporting

Please select the one below that is the best fit for your research. If you are not sure, read the appropriate sections before making your selection.

[x] Life sciences   [ ] Behavioural & social sciences   [ ] Ecological, evolutionary & environmental sciences

For a reference copy of the document with all sections, see nature.com/documents/nr-reporting-summary-flat.pdf

Life sciences study design

All studies must disclose on these points even when the disclosure is negative.

Sample size

No statistical methods were applied to predetermine the sample size for laboratory experiments.
Animal study: The sufficient sample size of each experiment is primarily based on similar experiments conducted in the past.
Deniger et al. Clin Cancer Res (2014). 3 mice per group
Wang et al. Front. in Immunol (2018). 3 mice per group.
Lu et al. EBioMedicine (2020). 5 mice per group.
Genomic analysis: We performed methylation arrays using 10^6 cells/sample, mRNA-seq using 10^6 cells/sample and Omni-ATAC-seq using 10^5/sample. Each treatment condition of individual cell lines was subject to genome-wide profiling once. The study design was similar to previous publications: Tsai et al. Cancer Cell (2012). Yi et al. Genomic Research (2017).

Data exclusions

No raw data were excluded from the analyses.

Replication

For cell lines or mouse experiments, at least three biological replicates were performed to verify the reproducibility of the experimental findings. All replication attempts were successful. For genomic analysis in cell lines or patient cohorts, we performed methylation arrays, mRNA-seq, and Omni-ATAC-seq once for each condition or for each patient.

Randomization

For mouse experiments, mice were randomly allocated into each treatment group. For cell experiments, cells from the same source of parental cell culture were assigned to each treatment condition randomly. The experiment was repeated at different times to minimize batch effects. For methylation analysis, samples under different treatment conditions were randomly allocated onto methylation arrays.

Blinding

For genomic analysis, the investigators who performed array hybridization, or library preparation and next-generation sequencing, were blinded to group allocation and treatment procedures. For microscopic imaging and cell counting, data collectors were blinded to treatment conditions. For animal experiment, blinding was not possible because individual groups of mice received different treatments over several cycles. The treatments and data recording were performed by the same investigator throughout.

Reporting for specific materials, systems and methods

We require information from authors about some types of materials, experimental systems and methods used in many studies. Here, indicate whether each material, system or method listed is relevant to your study. If you are not sure if a list item applies to your research, read the appropriate section before selecting a response.

Materials & experimental systems

n/a	Involved in the study	
[ ]	[x]	Antibodies
[ ]	[x]	Eukaryotic cell lines
[x]	[ ]	Palaeontology and archaeology
[ ]	[x]	Animals and other organisms
[ ]	[x]	Human research participants
[x]	[ ]	Clinical data
[x]	[ ]	Dual use research of concern

Methods

n/a	Involved in the study	
[x]	[ ]	ChIP-seq
[ ]	[x]	Flow cytometry
[x]	[ ]	MRI-based neuroimaging

Antibodies

Antibodies used

All antibodies and their supplier names, clone names, catalog numbers, dilutions used in the study are provided in the

	Supplementary Table 2.
Validation	The Research Resource Identifiers (RRIDs) for all antibodies used in this study are provided in Supplementary Table 2. Relevant publications or validation results for each antibody can be found through the RRID Portal, or on the manufacturer's website.

Eukaryotic cell lines

Policy information about cell lines

Cell line source(s)	Human lung cancer cell lines, A549, H1299, HCC827, PC-9, PC-9-IR, H2981, H157, H1792, H2170, a colorectal cancer cell line, HCT116, and 293T cells were obtained from the American Type Culture Collection (ATCC). CL1-0 and CL1-5 human lung adenocarcinoma cell lines were kindly provided by Prof. Pan-Chyr Yang at National Taiwan University College of Medicine (Chu, et al., 1997). CL1-0 and CL1-5 are not commercially available.
Authentication	Cell line authentications were performed using short tandem repeat (STR) analysis.
Mycoplasma contamination	All cell lines used in this study tested negative for mycoplasma contamination using EZ-PCR™ Mycoplasma Detection Kit (Biological Industries, 20-700-20).
Commonly misidentified lines (See ICLAC register)	None of the commonly misidentified lines were used in this study.

Animals and other organisms

Policy information about studies involving animals; ARRIVE guidelines recommended for reporting animal research

Laboratory animals	Six-week-old male NOD.Cg-PrkdcscidIl2rgtm1Wjl/SzJ (NSG) mice were used in this study. All mice were housed in an AAALAC accredited animal facility with a 12-hour dark/light cycle (8 a.m. to 8 p.m.) at a temperature between 20 and 24°C and a humidity between 50 and 70%.
Wild animals	This study did not involve wild animals.
Field-collected samples	This study did not involve samples collected from the field.
Ethics oversight	All mice experiments were approved by the NTU College of Medicine Institutional Animal Care and Use Committee (IACUC) (Protocol #20180077).

Note that full information on the approval of the study protocol must also be provided in the manuscript.

Human research participants

Policy information about studies involving human research participants

Population characteristics	Primary human peripheral blood mononuclear cells (PBMCs) were obtained from 6 healthy volunteers (4 males and 2 females) with no known medical conditions. The ages of the participants ranged from 21 to 45 years old. Informed consent was obtained from individual healthy donors before enrollment.
Recruitment	Healthy volunteers were recruited at National Taiwan University Hospital. The overview of the research project was provided orally or through recruitment flyers.
Ethics oversight	The study was approved by the Institutional Review Board (IRB) of National Taiwan University Hospital. We have complied with all ethical regulations.

Note that full information on the approval of the study protocol must also be provided in the manuscript.

Flow Cytometry

Plots

Confirm that:

- [x] The axis labels state the marker and fluorochrome used (e.g. CD4-FITC).
- [x] The axis scales are clearly visible. Include numbers along axes only for bottom left plot of group (a 'group' is an analysis of identical markers).
- [x] All plots are contour plots with outliers or pseudocolor plots.
- [x] A numerical value for number of cells or percentage (with statistics) is provided.

Methodology

Sample preparation	Ex vivo expanded γδ T cells were washed twice with PBS and stained with immunofluorescence antibodies targeting the surface markers, including γδTCR Vδ1, γδTCR Vδ2, CD27, CD69, NKG2D, TGF-β1, and CD107a. Subsequently, γδ T cells were fixed and permeabilized using Cytofix/Cytoperm solution (554714, BD Biosciences) for 20 minutes at 4°C for intracellular

	staining of cytokines, including IL-2, IL-10, IL-17A, IFN-γ, and TNF. Staining was performed at 4°C for 30 minutes in the dark. The samples were washed and fixed with 100 µl of 1X IOTest3 Fixative Solution (A07800, BECKMAN COULTER) per well for at least 10 minutes at 4°C. Cells were then resuspended in 300 µl PBS and analyzed on a flow cytometer.
Instrument	BD LSRFortessa flow cytometry (BD Biosciences).
Software	BD FACSDiva (BD Biosciences) is used for data acquisition. FlowJo software V10 (Tree Star) is used for the data analysis.
Cell population abundance	For annexing V apoptosis analysis, approximately 10,000 events of cancer cells were acquired for each sample.<br>For flow cytometric analysis of T cell polyfunctionality, about 30,000 singlet live γδ T cells were analyzed for individual cytokines.
Gating strategy	Live cells are gated by FSC-A and SSC-A. Positive and negative cell populations are defined based on the respective isotype controls.

☒ Tick this box to confirm that a figure exemplifying the gating strategy is provided in the Supplementary Information.
